# Shedding Light on Targeting Chronic Myeloid Leukemia Stem Cells

**DOI:** 10.3390/jcm10245805

**Published:** 2021-12-11

**Authors:** Mohammad Houshmand, Alireza Kazemi, Ali Anjam Najmedini, Muhammad Shahzad Ali, Valentina Gaidano, Alessandro Cignetti, Carmen Fava, Daniela Cilloni, Giuseppe Saglio, Paola Circosta

**Affiliations:** 1 Department of Clinical Biological Sciences, University of Turin, San Luigi University Hospital, 10043 Turin, Italy; mohammad.houshmand@unito.it (M.H.); muhammadshahzad.ali@unito.it (M.S.A.); carmen.fava@unito.it (C.F.); daniela.cilloni@unito.it (D.C.); paola.circosta@unito.it (P.C.); 2 Department of Hematology and Blood Banking, School of Allied Medical Sciences, Shahid Beheshti University of Medical Sciences, Tehran 1971653313, Iran; kazemi.alireza.1991@gmail.com (A.K.); anajmedni@gmail.com (A.A.N.); 3 Division of Hematology, A.O. SS Antonio e Biagio e Cesare Arrigo, 15121 Alessandria, Italy; valentina.gaidano@unito.it; 4 Division of Hematology and Cell Therapy, A.O. Ordine Mauriziano, 10128 Turin, Italy; alessandro.cignetti@unito.it

**Keywords:** chronic myeloid leukemia, leukemia stem cells, bone marrow microenvironment, treatment free remission

## Abstract

Chronic myeloid leukemia stem cells (CML LSCs) are a rare and quiescent population that are resistant to tyrosine kinase inhibitors (TKI). When TKI therapy is discontinued in CML patients in deep, sustained and apparently stable molecular remission, these cells in approximately half of the cases restart to grow, resuming the leukemic process. The elimination of these TKI resistant leukemic stem cells is therefore an essential step in increasing the percentage of those patients who can reach a successful long-term treatment free remission (TFR). The understanding of the biology of the LSCs and the identification of the differences, phenotypic and/or metabolic, that could eventually allow them to be distinguished from the normal hematopoietic stem cells (HSCs) are therefore important steps in designing strategies to target LSCs in a rather selective way, sparing the normal counterparts.

## 1. Introduction

The development of different generations of BCR-ABL1 tyrosine kinase inhibitors (TKIs) has led the overall survival (OS) of chronic myeloid leukemia (CML) patients to become almost similar to that of a control population without leukemia. However in most of the patients who discontinue the TKI therapy, a regrowth of the leukemic clone and a molecular recurrence of the disease can be observed and only approximately half of those who are achieving a very deep molecular response and therefore approximately only 15–20% of the entire CML population, can definitely and successfully suspend the therapy [1]. This is due to the persistence of leukemic stem cells that are able to survive in spite of the TKI therapy and may have the clonogenic capacity to resume the leukemic process once the TKI therapy has been interrupted. Therefore, alternative approaches for the elimination of TKI resistant leukemic stem cells are essential to increase the percentage of those who can reach a successful long-term treatment free remission (TFR) and possibly a definitive cure of the disease [2]. Considering that loss of long-term TFR can be due to the persistence of an even small number of leukemic stem cells (LSCs) showing a minimal, but extreme form of resistance to the TKI therapy, that may be due not to BCR-ABL1 mutations but mainly to BCR-ABL1 independent mechanisms, several studies attempting to identify possible metabolic and/or phenotypic differences between LSCs and normal hematopoietic stem cells (HSCs) are currently ongoing. Here we focus on the peculiar features of CML leukemic stem cells (CML LSCs) that have been so far discovered and that could be potentially useful as targets in designing therapeutic strategies aiming to eliminate in a rather selective way the residual LSCs while sparing their normal counterparts.

## 2. Metabolic Pathways Potentially Useful for Targeting CML LSCs

### 2.1. WNT Signaling Pathway

The WNT signaling pathway has been shown to have a significant role in the development of several organs including the hematopoietic system while perturbation of this crucial pathway sparks induction of various types of cancers. In the resting condition, the WNT-β catenin network forms a destruction complex including AXIN, adenomatous polyposis coli (APC), Casein kinase 1 (CK1) and Glycogen synthase kinase 3 (GSK3) and links to β-catenin, providing a binding site for the ubiquitin ligase that leads to β catenin degradation in the proteasome. By contrast, following attachment of WNT to the frizzled receptor (FR), GSK3, CK1, and AXIN bind Lipoprotein receptor-related proteins (LRP) and leave β-catenin free for nucleus localization where it interacts with transcription factors of the TCF/LEF family and promotes gene expression. It has been demonstrated that β-catenin is of paramount importance for self-renewal and long-term maintenance of both HSCs and LSCs [3]. After induction of BCR-ABL in β-catenin null mice, defect in self-renewal and in engraftment potential of CML LSCs has been observed and this shows that this pathway is essential for normal and leukemic stem cells survival [4]. It has been shown that BCR-ABL has direct contact with β-catenin and mediates the nucleus transition by making this protein more stable. Furthermore, expression of β-catenin increases with CML progression and is responsible for the increased self-renewal of CML progenitors in blast crisis [5]. In the bone marrow niche, mesenchymal cells may interact with CML LSCs through the WNT/β- catenin pathway enhancing their proliferation. Therefore, increased expression of β-catenin can be seen as a form of resistance of LSCs allowing their survival [6,7] and a combination of WNT/β-catenin inhibitors and TKI could potentially help to get rid of CML LSCs. However, unfortunately this approach has been demonstrated to be too toxic also for normal HSCs as well as for the stem cells of other organs and, at least for the moment, has been abandoned [8,9].

### 2.2. Hedgehog Signaling Pathway

The Hedgehog signaling pathway which is essential for hematopoiesis, is deregulated in several solid tumors. It is initiated through binding of hedgehog (Hh) ligands (Sonic Hh, Indian Hh, and Desert Hh) to a seven transmembrane receptor called Patched (Ptch). After the consequent activation of the Smoothened protein (Smo) by Ptch, Glioma-Associated Oncogene Homolog (Gli) family transcription factors are activated, which are able to transcribe target genes such as Gli1, Ptch1, bcl2, Cyclin D, and MYC [10]. In CML high mRNA expression of Hedgehog cascade related proteins underlines the role of this pathway in driving leukemogenesis [11]. ABL kinase is not needed for the salvation of this cascade [12]. Whereas studies suggest that Smo targeting does not affect the engraftment potential and the fate decision of normal HSCs, it has been shown that it may potentially reduce the engraftment ability and the colony formation of CML LSCs. Therefore, hitting this pathway should selectively affect LSCs but not normal HSCs [12,13]. Indeed, exposure of CML cells containing both wild type BCR-ABL and BCR-ABL1 mutated T315I cells to an Smo inhibitor led to the purging of the mutated clone [14]. Another study demonstrated that the use of Hedgehog inhibitors not only propels CML LSCs into cycling condition but also restores their susceptibility to TKI [13,15]. Again, however clinical trials testing the usefulness of this approach have shown that Hh pathway inhibitors are too toxic and have been finally abandoned.

### 2.3. PI3K-AKT Pathway

The known participation of the Phosphatidylinositol-3-kinase (PI3K) signaling pathway in the maintenance and function of normal HSCs drove the attention on the possible role of this cascade in the LSC population. By phosphorylation of phosphatidylinositol (3,4)-bisphosphate (PIP2) by PI3K and formation of phosphatidylinositol-3,4,5-trisphosphate (PIP3), Pyruvate Dehydrogenase Kinase 1 (PDK1) is recruited and associated to PIP3 and phosphorylates AKT, that subsequently activates mTORC1 and phosphorylates the Forkhead box O (FOXO) transcription factors family [16]. It has been shown that this signaling pathway is activated by BCR-ABL1 and so, in the Ph-positive cell population, it may be more specific with respect to the WNT and Hedgehog signaling pathways. When the BCR-ABL TK activity is on, AKT phosphorylates FOXO transcription factors and does not allow their shift to the nucleus, but TKIs blocking BCR-ABL1 TK activity can promote FOXO nucleus relocalization, restoring their transcriptional activity. Expression of BCL6, that is considered essential for the survival of CML stem cells, and also ATM and CDKN1C, is enhanced by the FOXO transcriptional activity [16,17]. Inhibition of mTORC1 did not show an evident effect on CML LSCs, but inhibition of PI3K can restore the vulnerability of CML LSCs to TKIs [18].

### 2.4. JAK-STAT Pathway

In addition, the JAK-STAT pathway plays an important role in CML, but in a strong association with BCR-ABL1 kinase activity. Indeed, STAT1, STAT3, and STAT5 can be activated by BCR-ABL1 directly or indirectly through JAK2 induction and activation by BCR-ABL1. JAK2 activation can be also stimulated by growth factors produced by the mesenchymal cells of the hematopoietic bone marrow niche [19,20]. It has been shown that inhibition of the JAK2 by ruxolitinib may reduce the level of BCR-ABL1 protein and may help to overcome resistance [21]. It was also seen that the combination of Imatinib + INFγ is able to decrease the phosphorylation of STAT5, but it increases the phosphorylation of STAT1, an up-regulator of the survival hint induced by BCL6, clearly delineating another potential pitfall of imatinib and of TKI therapy in general [22]. In line with these concepts, the use of ruxolitinib (a JAKs inhibitor) together with nilotinib fruitfully in vitro showed activity in suppressing the CML LSC population, without affecting the HSCs. However clinical trials testing this strategy were unsuccessful. It has also been reported that STAT3 dysregulates CML LSC metabolism, and its inhibition may eradicate these resistant cells [23]. Meanwhile, glitazones an antidiabetic drug by activating peroxisome proliferator-activated receptor-γ (PPARγ) reduces expression of STAT5 that might lead to the elimination of the CML LSC population [24].

**Figure 1:**
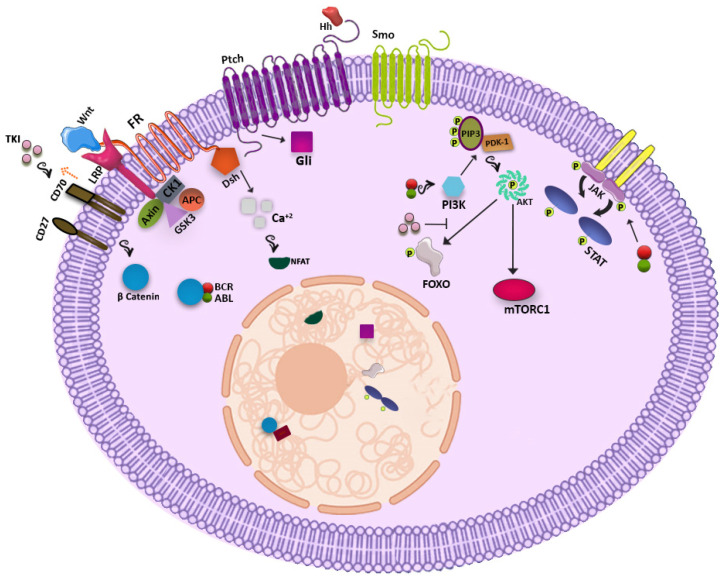
Possible signaling pathways in CML LSCs.

Possible signaling pathways are shown in Figure 1.

### 2.5. Other Players

#### 2.5.1. Blk

It has been shown that Blk as a tyrosine kinase protein has a diminished expression in CML LSCs in contrast to normal cells. Although Blk is regarded as a tumor suppressor, in CML LSCs, via upregulation of p27, BCR-ABL1 downsizes the expression of this protein by modulation of c-myc and Pax5. Meanwhile, overexpression of Blk in CML LSCs inhibits the self-renewal and increases the apoptosis rate, while Blk knock-down does not interfere with the regular HSC activity [25].

#### 2.5.2. EZH2

EZH2 as part of the PRC2 complex mediates repression of various genes by trimethylation of histone H3 (H3K27me3). Amplification of EZH2 in CML LSCs and reduction after TKI therapy shows its engagement in the pathogenesis of CML and its dependency on BCR-ABL1 TK activity. It has also been reported that EZH2 inhibitor increases the possibility of CML LSC eradication while sparing the normal HSCs. This effect is enhanced by the combination of TKI and EZH2 Inhibitor [26].

#### 2.5.3. Fap-1

Another pathway associated with resistance to elimination is the enhanced expression of Fap-1 in CML LSCs. Fap-1 with its phosphatase activity blocks Fas mediated apoptosis and also stabilizes β-catenin by targeting its inhibitor Gsk3β. Fap-1 activity is accompanied by persistence of CML stem cells and Fap-1 inhibition promotes TKI response and hampers the progression of leukemic cells [27].

#### 2.5.4. HIF-1

Hypoxia inducible factor (HIF), constituted of α and β subunits, increases when oxygen concentration is low in order to facilitate the cell adaptation to this new environment. HIF-1 as a transcription factor has a crucial role in regulating survival, proliferation, and maintenance of CML LSCs. Cheloni et al., posited that using acriflavine, an HIF-1 inhibitor, significantly affects the fate of CML cells by c-MYC down regulation and decrease of stemness genes like NANOG, SOX2, and OCT4. As CML LSCs are more dependent on HIF-1 than normal HSCs, the combination of an HIF-1 inhibitor with TKI could represent a new form of strategy to target resistant CML LSCs that reside in the hypoxic region [28,29].

#### 2.5.5. PML

The promyelocyte leukemia protein (PML), by attending in the formation of PML-nuclear bodies (PML-NBs), acts as a tumor suppressor and transcription factor and plays a pivotal role in apoptosis and senescence of normal cells [30]. The PML gene is already well known because of its involvement in the t(15;17) translocation that causes the fusion of PML with retinoic acid receptor alpha (RAR-alpha) in acute promyelocyte leukemia (APL), determining a differentiation arrest [31]. Besides, up-regulation of PML in CML LSCs may hamper the cycling of these cells and cause a decrease in their sensitivity to TKIs. It has been shown that targeting PML in CML cells by arsenic trioxide (As_2_O_3_) leads to PML degradation and triggers cycling of these quiescent cells. This strategy may promote the exhaustion of the CML LSCs restoring their sensitivity to the TKIs [32].

#### 2.5.6. PP2A

Protein phosphatase 2 A (PP2A), a serine/threonine phosphatase which is composed by the scaffold (A), regulatory (B), and catalytic (C) subunits, has a role in directing β-catenin pathway, programmed cell death and cell cycle progression [33]. Until now our knowledge about the role of PP2A in CML LSCs has been limited to its tumor inhibitory effect. In CML this protein is regulated by SET protein activity, and enhancement of SET during the progression of CML from chronic to more advanced phases of the disease determines the downregulation of PP2A. It has been shown that in the LSC population, PP2A reduction provides a stimulus for self-renewal of leukemic cells. Restoring its activity could therefore be useful to decrease the LSC pool [34]. Various isoforms of PP2A, however are present and while some of them play a suppressive role in many cancers, other isoforms can act differently [33]. Recently, however, Lai et al. demonstrated that inhibition of PP2A and TKI may efficiently suppress CML LSCs [35].

#### 2.5.7. ALOX5

ALOX5 encodes 5-lypoxygenase (5-LO) that converts arachidonic acid into leukotrienes and is involved in inflammatory condition and cancer development [36]. Targeting ALOX5 hampers the differentiation, the function, and the survival of CML LSCs, while normal HSCs remain uninfluenced. Zileuton (5-LO inhibitor) impairs CML LSC development [37], but although the oncogenicity of ALOX5 in the mouse model seemed to be compelling, in CML patients it has a low expression and the use of a 5-LO inhibitor does not show particular consequences [38].

#### 2.5.8. SIRT1

Sirtuin 1 (SIRT1) is a histone deacetylase that regulates gene expression, metabolic activity and aging within cells [39]. SIRT1 overexpression in primary CML cells deacetylates many transcription factors including P53, Ku70, and FOX01. This genetic modification promotes drug resistance, survival, and propagation of the leukemic fraction [40,41]. SIRT1 targeting in CML LSCs, both by inhibition or knock-down, enhances acetylation of P53 which gives rise to apoptosis and reduction of their growth [42]. So, applying the combination of TKI and SIRT1 inhibitor maybe a potential approach to tackle leukemogenesis.

Considering the role of different molecules in supporting CML LSC survival and proliferation, many clinical trials have been designed to target these players and are summarized in Table 1.

#### 2.5.9. microRNAs

microRNAs (miRNA), a class of non-coding RNAs, in physiological conditions regulate various cellular process such as differentiation [43], proliferation, metabolism, and apoptosis through mechanisms of repression of translation and mRNA cleavage [44]. Dysregulation of miRNAs is a crucial determinant in the pathogenesis of multiple cancers [45] and some data suggest a possible active involvement of several microRNAs also in CML LSC resistance, self-renewal, and maintenance processes. miR-126 is considered to be the regulator of dormancy of CML LSCs and HSCs. Active BCR-ABL1 phosphorylates SPRED1 which in turn depletes the amount of mature miR-126. This depletion should be compensated for by an external source to keep up stemness features. In bone marrow niche endosteal Sca-1+ cells can provide a high amount of miR-126 through extracellular vesicles. Conversely, the TKI therapy can reverse the mechanism of miR-126 inhibition and elevate the mature miR-126 level, contributing to maintaining the CML LSCs. Considering this mechanism, decreasing the activity of miR-126 may sensitize LSCs to TKI and facilitate their elimination [46]. On the other hand, the parallel enhanced activity of the JAK-STAT pathway as described before may induce ADAR1 expression and activity, an enzyme that converts adenosine to inosine and regarded as a post transcriptional regulator able to control the stability of mRNA and miRNAs. ADAR1 can in turn impair the biogenesis of mir-let7, a tumor suppressor, and modulate the self-renewal of CML LSCs [47]. Exposure of primitive CML cells to imatinib is associated with an elevation of miR- 21 and may culminate in TKI resistance. So, depletion of miR-21 through amplification of PDCD4 and PTEN by interrupting the PI3K/AKT pathway may restore the sensitivity of CML LSCs to TKI [48]. In another experiment mir-30a downregulation following imatinib treatment allowed autophagy related proteins Beclin1 and ATG5 to push LSCs towards resistance [49]. Therefore, although, the role of long non-coding RNAs in the CML LSC fraction have not been totally defined, participation of mi-RNAs supports a role for these non-coding RNAs in LSC persistence.

## 3. The Environment

### Bone Marrow Niche

At the time of introducing the term “niche” by Schofield in 1978, many experiments had been accomplished to uncover the mysterious role of this spatial structure in the normal and leukemic state. This microenvironment not only encompasses stromal cells, HSCs, endothelial cells, and neural cells but also interactions, secretions of varied molecules, and signaling pathways are included in the definition [49]. As normal hematopoiesis hinges upon the equilibrium between osteoblastic niche for preserving stemness and vascular niche for differentiation strategy, in the leukemic condition, an erratic contest between LSCs and HSCs leads to creation of the leukemic niche [50]. It has been postulated that expression and signaling of CXCR4 are modulated by the level of BCR-ABL [51] and higher expression of BCR-ABL both in mRNA and protein levels in CML stem cells [52] may cause downregulation of CXCR4 and release of CML stem cells into the peripheral blood. Simultaneously, as mentioned above, CD26 by disruption of CXCL12 as an accompaniment is deeply involved in this process [53]. In the interim, enhancement of CXCR4 by imatinib may be mediated by suppression of BCR-ABL, triggering the homing process [51]. This lodgment into the bone marrow niche induces CML stem cells to become quiescent, chemoresistant, and constitute the leukemic stem cell reservoir [51,54]. In addition, CML LSCs in contrast with their normal counterpart are not dependent on VLA-4 and VLA-5 for homing. It has been reported that E and L-selectin and related ligands such as CD44 play an important role in CML LSC homing. Meanwhile, TKI exposure may lead to adhesion of CML LSCs to stromal cells through N-cadherin that may result in activation of the B-catenin pathway and protection against TKIs [50]. It has been demonstrated that CML cells by secretion of exosome containing amphiregulin, switch on epidermal growth factor (EGFR) pathway in stromal cells. This interface increases the secretion of IL-8 and increases cell adhesion and survival of leukemic cells [55]. Besides, multiple mechanisms amalgamate to promote evasion of CML LSCs from destruction in the bone marrow niche by common TKIs. For instance, higher expression of BMPR1b in more primitive CML cells and activation via BMP4 through both the paracrine and autocrine loop boosts expression of TWIST1, a contributor to drug resistance [56]. Other studies indicated that the presence of stromal cells whether by reduction of ROS or production of FGF2 support chemoresistance and act as the sanctuary of CML LSCs [57,58,59]. However, we should take note that these mesenchymal stem cells are not the precedent ones due to the many genetic changes in dealing with the leukemic niche [60,61]. Meanwhile, participation of the adipose tissue in the treatment circumvention reminds us that endosteal and vascular milieus are only the tip of the iceberg. CML LSCs in the adipose niche consume free fatty acid as a source of energy, and by enhancing lipolysis have a hand in progression, drug resistance, and body weight loss [62]. Aside from these scenarios in niches, numerous signaling pathways in BCR-ABL dependent and independent manner are ongoing and should be taken into account in baring the facts of CML LSC persistence.

## 4. Phenotypic Differences

The CML LSCs reside in the CD34^+^/CD38^−^ fraction as the normal ones [63], and a good CD marker useful to distinguish them is expected to be preferentially expressed in one of the two populations, the leukemic or the normal one. Indeed, different studies have identified several surface markers potentially able to allow the recognition of CML LSCs, although in some cases these markers are simply expressed by both populations in a different or asynchronous way. In this category we can insert CD33, that is expressed by normal HSCs as well as by blasts of acute myelogenous leukemia (AML), but not at a high level as in CML LSCs [64]. Similarly, CD36, a scavenger receptor, shows a low expression on normal HSCs, but it is highly expressed in CML LSCs [65]. Other markers such as CD25, CD26, and IL-1RAP that are also highly expressed by CML LSCs appear more suitable. In the CD34^+^/CD38^−^ fraction, CD25 (IL-2Rα) is exclusively expressed by CML LSCs as we cannot detect it on normal HSCs. A low amount of CD25 on the surface of normal HSCs becomes however detectable in the CD34^+^/CD38^+^ fraction [66]. Interleukin 1 receptor accessory protein (IL-1RAP) is not expressed by HSCs and theoretically can be used to detect CML LSCs. According to some studies however not all the IL-1RAP expressing cells are Ph^+^ LSCs [67]. Furthermore, expression of IL-1RAP is much more pronounced in the advanced phases of the disease with respect to that of the CML LSCs present in the chronic phase [68], making this marker more suitable for resistant cases rather than to increase the percentage of those reaching a successful TFR, who are per definition in the category of very good responders. On the contrary, CD26 (DPP4, a serine exopeptidase with a cleavage activity against diverse substrates and able to demolish SDF1 on the SDF1-CXCR4 axis contributing in this way to the release of the CML LSCs into the peripheral blood) [53,69] is aberrantly expressed by CML LSCs/progenitor cells and has no expression on normal HSC/progenitor cell population [70]. It has been reported that CD26^+^ cells are detectable in newly diagnosed and resistant CML patients and also in those who are in TFR [71]. In a normal context, CD26 is expressed mainly by activated T cells and has a relation with the proliferation of these cells. In certain other conditions including autoimmune diseases, lung adenocarcinoma, hepatocellular carcinoma, B-chronic lymphoblastic leukemia, and T acute lymphoblastic leukemia, enhanced expression of CD26 is detectable, but previous reports claimed that inhibition of CD26 does not suppress the activation and cytotoxic effect of T cells [68,69,72]. Until now, among the suggested markers, CD26 can therefore be considered to be the best option for CML LSC detection and targeting. Indeed, in a recent study a monoclonal antibody against CD26 called Begelomab (used in clinics for the treatment of acute graft versus host disease, aGvHD) [70] has been conjugated to a PEGylated liposome. Using this immunoliposome (IL), it was demonstrated that LSCs from primary CML samples could be selectively targeted, but not their normal counterparts. As Begelomab alone had no toxicity in treated cells, the immunoliposome was used as a carrier for venetoclax, a BCL2 inhibitor already demonstrated as being efficacious in eliminating CML LSCs. This anti CD26 immunoliposome carrying venetoclax not only eliminated CD26^+^ cells but also reduced the drug concentration that was required to induce apoptosis in leukemic cell lines. This strategy consisting in recognizing specific antigens on the surface of the LSCs, demonstrates therefore higher efficiency in comparison to free drug administration and can also decrease the toxicity due to off-target effects of the drugs. In addition to CD26, CD93 has been reported to be another antigen for targeting. It has been reported that CD93 has a role in the self-renewal and proliferation of the CML LSCs and CD93 expressing LSCs showed resistance to TKIs [73,74].

The phenotypic characteristic of CML LSCs is summarized in Table 2.

## 5. Conclusions and Future Perspectives

Different durations of deep molecular response (DMR) have been seen to be associated with different probabilities of achieving TFR. However, even in the presence of the theoretically best conditions to maintain a long TFR, a threshold of 70–75% for successful TFR cannot be exceeded. This has been ascribed to the presence of LSCs resistant to TKI therapy because of the BCR-ABL1 independent mechanisms of resistance, that can persist even in patients in DMR and that, on discontinuation of the TKI therapy, can resume the leukemic process. Time to loss TFR varies from patient to patient and might be germane to the heterogeneity in the LSC population. It has been hypothesised that patients with a faster LSC cell growth rate might face a faster molecular relapse. As reviewed above, several phenotypic and biological characteristics can theoretically distinguish between CML LSCs and their normal counterparts and can therefore represent potential targets to hit these TKI resistant LSC subclones, increasing the percentage of those able to achieve a long-term TFR, and that after five years of duration can be regarded as a definitive cure of the disease, at least from an operational point of view. Currently, however, many attempts of trying to achieve this goal with various forms of combination therapy have failed. Indeed, in most cases this is not due to lack of efficacy, but to a level of toxicity that, if acceptable for patients in critical clinical situations, is unacceptable for patients that are doing well and have a normal life expectancy and a good quality of life even continuing TKI therapy. Therefore, in order to offer a clinical advantage, all that we have learned in terms of biology of the LSCs and about their differences with respect to those of normal HSCs, requires to be translated into therapeutical approaches that are mild and safe for patients. These kinds of approaches cannot be easily tested when using new drugs, because generally new treatments are first offered to patients without other therapeutical options, where both efficacy and toxicity are tested in a totally different scenario with respect to that of patients already having good response and in complete remission. Possible options can be represented by testing these new approaches only when absolutely needed as in the patients who have failed the first attempt to reach TFR and who, because of side effects or for the strong wish to discontinue the therapy in any case, may accept the risk of mild additional toxicity, at least for a limited period of time. In the end only time will tell us whether the investment in studying CML LSCs will be of clinical value, as we all really hope.

## Figures and Tables

**Table 1:** Clinical trials that used non-TKI agents for the treatment of CML.

CML Clinical Trial with New Therapeutic Agents (Non-TKIs)					
Generic Name	Brand Name	Clinical Trial Identifier	Target	Start Date	Status
Sirolimus	Rapamune	NCT00101088	mTOR inhibitors	10-Jan-05	Terminated
Sorafenib	Nexavar	NCT00661180	Multikinase inhibitors, VEGF/VEGFR inhibitors	18-Apr-08	Completed
Sunitinib	Sutent	NCT00387426	Multikinase inhibitors, VEGF/VEGFR inhibitors	13-Oct-06	Completed
Ruxolitinib	Jakafi	NCT02253277	JAK/STAT inhibitors	1-Oct-14	Completed
Axitinib	Inlyta	NCT02782403	Multikinase inhibitors, VEGF/VEGFR inhibitors	25-May-16	Terminated
Ibrutinib	Imbruvica	NCT03267186	BTK inhibitors	30-Aug-17	Ongoing
Midostaurin	Rydapt	NCT02115295	Multikinase inhibitors	16-Apr-14	Ongoing
PRI-724	-	NCT01606579	Wnt/β-catenin inhibitors	25-May-12	Completed
BP1001	-	NCT02923986	Grb2	5-Oct-16	Withdrawn
Tipifarnib	Zarnestra	NCT00040105	Farnesyl transferase	21-Jun-02	Completed
Lonafarnib	SCH66336	NCT00047502	Farnesyl transferase	9-Oct-02	Completed
Rapamycin	Sirolimus	NCT00780104	mTOR	27-Oct-08	Completed
RAD001	Everolimus	NCT01188889	mTOR	26-Aug-10	Withdrawn
Panobinostat	LBH589	NCT00451035	Histone deacetylase	22-Mar-07	Terminated
Azacytidine	Vidaza	NCT03895671	Hypomethylating agents	29-Mar-19	Ongoing
MK-0457	Tozasertib	NCT00405054	Aurora kinase pathway inhibitors	29-Nov-06	Terminated
Venetoclax	Venclexta	NCT02689440	BCL-2 inhibitors	24-Feb-16	Ongoing
Temsirolimus	Torisel	NCT00101088	mTOR	10-Jan-05	Terminated
Abemaciclib	Verzenio	NCT03878524	CDK 4/6 inhibitors	18-Mar-19	Ongoing
Alemtuzumab	Lemtrada/campath	NCT00626626	CD52 monoclonal antibodies	29-Feb-08	Terminated
Bevacizumab	Avastin	NCT00023920	VEGF/VEGFR inhibitors	27-Jan-03	Terminated
Blinatumomab	Blincyto	NCT02790515	Miscellaneous antineoplastic	6-Jun-16	Ongoing
Ipilimumab	Yervoy	NCT00732186	Anti-CTLA-4 monoclonal antibodies	11-Aug-08	Withdrawn
Nivolumab	Opdivo	NCT02011945	Anti-PD-1 monoclonal antibodies	16-Dec-13	Completed
Rituximab	Rituxan	NCT03455517	Antirheumatics, CD20 monoclonal antibodies	6-Mar-18	Terminated

**Table 2:** Phenotypic characteristic of CML LSCs.

Marker Name	CD Name	CML Cells		Normal Cells		References
		LSCs	Progenitor Cells	Stem Cells	Progenitor Cells	
		CD34^+^/CD38^−^	CD34^+^/CD38^+^	CD34^+^/CD38^−^	CD34^+^/CD38^+^	
**IL-2Rα**	CD25	+	+/−	−	+/−	[75]
**DPPIV**	CD26	+	+/−	−	−	[53]
**Siglec-3**	CD33	+	+	+/−	+/−	[76]
**SCARB3**	CD36	+	+	+/−	+	[65]
**Pgp-1**	CD44	+	+	+	+	[76]
**IAP**	CD47	+	+	+	+	[77]
**Campath-1**	CD52	+	−	+/−	+/−	[76]
**MXRA4 (C1qR1)**	CD93	+	+/−	+/−	+/−	[73,76]
**MIC2**	CD99	−	unknown	+	+	[78]
**SCFR (KIT)**	CD117	+	+	+	+	[76]
**IL-3Rα**	CD123	+	+	+	+	[76]
**AC133**	CD133	+	+/−	+	+	[76]
**BST1**	CD157	+	+	+	+	[76]
**CLL-1**	CD371	−	+	−	+	[76]
**TIM-3**	-	−	unknown	+/−	+/−	[79]
**IL-1RAP**	-	+	+	−	+	[68]

CML LSC: Chronic myeloid leukemia stem cells; IL-2Rα: Interleukin-2 receptor alpha; DPPIV: Dipeptidyl peptidase IV; Siglec-3: Sialic acid-binding immunoglobulin-type lectin-3; SCARB3: Mast/stem cell growth factor receptor; Pgp-1: Phagocytic glycoprotein-1; IAP: Integrin associated protein; SCFR: Stem cell factor receptor; IL-3Rα: Interleukin receptor subunit α; CLL-1: C-type lectin-like molecule-1; TIM-3: T-cell immunoglobulin mucin-3; IL-1RAP: Interleukin-1 receptor accessory protein.

## Data Availability

Not applicable.
